# Determinants of Rice Grain Quality: Synergistic Roles of Genetics, Environment, and Agronomic Practices

**DOI:** 10.3390/ijms27073088

**Published:** 2026-03-28

**Authors:** Liqun Tang, Honghuan Fan, Junmin Wang, Kaizhen Zhong, Hong Tan, Fuquan Ding, Ling Wang, Jian Song, Mingli Han

**Affiliations:** 1 Institute of Crops and Nuclear Technology Utilization, Zhejiang Academy of Agricultural Sciences, Hangzhou 310021, China; liquntang2013@126.com (L.T.); xixi615@163.com (H.F.); wangjm917@sina.com (J.W.); zkz0804@163.com (K.Z.); sammy_cn@163.com (H.T.); 2 Huzhou Academy of Agricultural Sciences, Huzhou 313000, China; dingfuquan007@163.com (F.D.); 2019101155@njau.edu.cn (L.W.)

**Keywords:** rice (*Oryza sativa* L.), grain quality, genotype-environment-management (G × E × M) interaction, water management, nitrogen fertilization

## Abstract

Rice (*Oryza sativa* L.) grain quality is a critical determinant of market value, consumer acceptance, and nutritional security. This multifaceted trait is governed by the dynamic interaction of genotype (G), environment (E), and management practices (M). In this review, we synthesize recent advances in understanding these multifaceted determinants. We first delineate the genetic architecture, emphasizing key genes and quantitative trait loci (QTLs) such as *Wx*, *ALK*, *Chalk5*, and the *GS3/GW* families, which control starch composition, gelatinization temperature, chalkiness, and grain dimensions, forming the foundational blueprint for quality potential. We examine how this genetic potential is influenced by environmental factors, focusing on the detrimental impacts of abiotic stresses, particularly high temperatures during grain filling and drought, which impair milling yield, increase chalkiness, and modify starch and protein profiles. Furthermore, we discuss how optimized agronomic strategies—including precision water management (e.g., alternate wetting and drying), balanced nitrogen fertilization, and targeted micronutrient (e.g., silicon) application—can mitigate these adverse effects and potentially improve specific quality parameters. Post-harvest handling is identified as the final determinant of product quality. We conclude that achieving high and stable rice quality under climate variability requires an integrated G × E × M approach. Prospects include next-generation breeding for climate-resilient quality, precision agronomy guided by real-time sensing, synergistic soil health management, and the integration of systems biology with digital agriculture to design sustainable, high-quality rice production systems.

## 1. Introduction

Rice (*Oryza sativa* L.) is a staple food for more than half of the world’s population, making its yield and quality essential to global food security [1]. While significant efforts have historically concentrated on increasing grain production, grain quality has emerged as an equally vital trait governing consumer preference, market value, and nutritional health. Rice grain quality is not a singular attribute but a complex, multifaceted concept encompassing four primary categories: milling quality, appearance quality, cooking and eating quality, and nutritional quality [2]. The inherent complexity of these traits arises from the intricate interaction between the plant’s genotype and the environment in which it is cultivated. The genetic control of quality traits has been progressively decoded with the advancement of molecular biology. Major quantitative trait loci (QTLs) and genes regulating key quality parameters have been identified and cloned. For instance, the *Waxy* (*Wx*) gene encodes granule-bound starch synthase (GBSS), which is the determinant of amylose content, a core factor influencing eating and cooking quality [3]. Similarly, genes such as *Chalk5*, which encodes a vacuolar H+-translocating pyrophosphatase, are associated with chalkiness formation. That is, an appearance quality defect [4].

However, the expression of these genetic potentials is profoundly modulated by environmental factors and agronomic practices. Climate change-induced abiotic stresses, such as high temperature during the grain-filling period, significantly increase chalkiness and reduce milling recovery, thereby compromising appearance and commercial quality [5]. Conversely, post-anthesis moderate heat stress has been found to alter the expression of starch synthesis genes, resulting in a decrease in amylose content and potentially softer texture [6]. Water management, particularly alternate wetting and drying (AWD), can enhance eating quality by reducing amylose content and protein content [7]. Nutrient management, particularly nitrogen fertilization, plays a dual role; while essential for yield, excessive application often increases protein content at the cost of eating quality [8]. Recent studies have emphasized the role of silicon fertilization in reducing heavy metal accumulation and improving grain nutritional safety [9]. Furthermore, harvest timing is crucial, as it directly affects moisture content and milling quality [10]. Post-harvest handling, including drying, storage, and milling, constitutes the final critical phase determining the quality reaching consumers. Inadequate drying conditions can cause fissured kernels, drastically reducing head rice yield [11]. Therefore, achieving high and stable rice quality demands an integrated approach that combines superior genetics, favorable environmental conditions and careful post-harvest practices. This review aims to synthesize recent advances in understanding the determinants of rice grain quality, focusing particularly on new genetic insights, the impact of changing climate conditions, and innovative agronomic strategies. By integrating findings from molecular physiology, genomics, and agronomy, we aim to provide a comprehensive framework for guiding the breeding and cultivation of high-quality rice sustainably.

## 2. Determinants of Rice Grain Quality

### 2.1. Genetic Influences on Grain Quality

**Figure 1:**
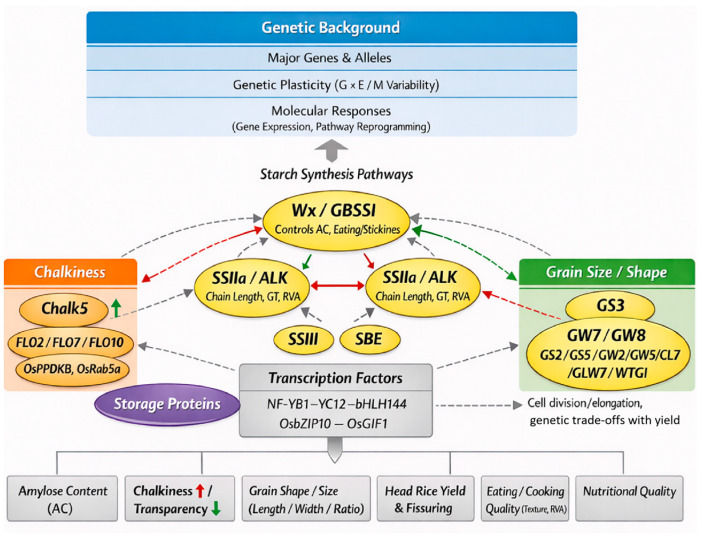
Genetic regulatory network of key determinants influencing rice grain quality. Legend: (1) Solid green arrows (+) indicate positive regulation or activation. (2) Solid red arrows indicate negative regulation or inhibition. (3) Dashed green arrows (+) indicate indirect regulation or activation. (4) Dashed red arrows (+) indicate indirect regulation or activation. (5) Dashed gray arrows indicate indirect associations, pleiotropic effects, genetic trade-offs, or genotype × environment (G × E) sensitivity.

Rice grain quality can be conceptualized as a three-tier structure: (i) the genetic background, which sets the upper limits and baseline phenotypes via major genes and allele combinations; (ii) genetic plasticity, which reflects variability across environments and management; (iii) molecular responses, which involves gene expression and metabolic pathway reprogramming for short-term phenotypic adjustments (Figure 1). Framing the varietal foundation as the starting point contextualizes subsequent environmental and management effects within an interpretable genetic framework [12,13,14].

Numerous genes influence the quality of rice (Table 1). Eating and cooking quality are primarily regulated by starch synthesis pathways. The *Wx* gene (encoding GBSS) regulates amylose content, serving as the major determinant of eating quality and stickiness in japonica/indica types [15,16]. Secondary enzymes like Starch Synthase IIa (SSIIa) are encoded by Alkali degeneration gene (*ALK*).

Starch Synthase III (SSIII) and Starch Branching Enzyme (SBE 99) influence starch chain-length distribution, gelatinization temperature and Rapid Visco Analyzer (RVA) profiles [17,18,19]. Functional validation and natural variation studies reveal that *Wx* alleles significantly alter amylose content and exhibit differential heat sensitivity, resulting in quality degradation under high night temperature or grain-filling heat stress [20,21]. Recent functional genomics has identified floury endosperm genes and Heat Shock Protein (HSP)-related proteins in endosperm starch biosynthesis and heat tolerance, emphasizing protein stability’s role in maintaining starch synthesis under heat stress [22].

Grain shape and size are regulated by loci such as Grain Size 3 (*GS3)* and *GW7* (also called *GL7*)/*GW8* (also called *OsSPL16*), which often trade off with yield traits in breeding [23,24]. Chalkiness arises from abnormal endosperm cell structure and starch granule arrangement; genes such as *Chalk5* directly influence endosperm redox and protein folding environments, altering transparency [4]. Head rice yield and grain fissuring are influenced by pericarp structure, 1000-grain weight, and starch-protein complexes, correlating with drying and processing sensitivity [25]. Beyond structural enzyme genes, transcription factor complexes directly regulate key enzyme expression, altering quality phenotypes. For example, the NF-YB1–YC12–bHLH144 complex activates *Wx* to regulate amylose content [26], while *OsbZIP10* influences grain shape and eating quality via regulation of Grain Incomplete Filling 1 (*OsGIF1*), which catalyzes the breakdown of sucrose into glucose and fructose in developing grains [27]. These modules exhibit transcriptional-translational coupling, intersecting with hormone signaling and heat/oxidative stress responses [28].

Increasing evidence shows that effects of genes such as *Wx* and *SSIIa* are not constant—they exhibit varying sensitivity to high temperature and drought [29]. Certain *Wx* alleles reduce amylose content or alter molecular structure under heat, changing gelatinization behavior and sensory scores [3,21]. Nitrogen can indirectly lower eating quality by increasing grain protein (interacting with amylose content/Rapid Visco Analyzer), with effects varying by genotype, emphasizing the need to incorporate environmental/management responsiveness in breeding targets [30].

**Table 1:** Selected Genes Associated with Rice Quality and Their Functions.

Quality Traits	Gene Name	Gene Function	Reference
Amylose content	*Wx*	Regulates the expression of starch synthase to influence the amylose content in rice grains	[15]
Chalkiness	*Chalk5*	Positively regulates the chalk grain rate of rice	[4]
	*FLO2*	Plays a crucial role in regulating grain size	[31,32]
	*FLO7*	Exerts a crucial regulatory function during the development of powder cells and the synthesis of peripheral endosperm starch	[33]
	*FLO10*	Encodes a P-type PPR (Pentatricopeptide Repeat) protein, which plays an important role in endosperm development	[34]
	*OsPPDKB*	Plays a crucial role in the starch metabolism and structure of rice endosperm	[22]
	*OsRab5a*	Plays a crucial role in the localization of glutelin mRNA to the cortical endoplasmic reticulum; its deficiency leads to quality issues	[35]
Gelatinization temperature	*ALK*	Encodes the soluble starch synthase II, which controls the gelatinization temperature of rice	[17,36]
Aroma	*BADH2*	Encodes a betaine aldehyde dehydrogenase, which regulates aroma by catalyzing betaine aldehyde and 4-aminobutyraldehyde	[37]
Storage Proteins	*OsGluA2*	Increases grain storage proteins and total amino acids, upregulating protein content in grains and enhancing the number and size of Protein Body II (PB-II), ultimately improving nutritional quality	[38]
	*qPC1*	Encodes a putative amino acid permease, OsAAP6, which positively regulates grain protein content	[39,40]
Grain size	*GS2*	Regulates cell elongation and division, influencing grain size and weight in rice	[41,42]
	*GS3*	A major QTL for grain weight and length in rice, with pleiotropic effects on grain width and grain filling	[43,44]
	*GS5*	Encodes a serine carboxypeptidase that acts as a positive regulator of grain size in rice	[45,46]
	*GS9*	Functions as a transcription factor that regulates grain morphology through the modulation of cell division	[47]
	*GW2*	Encodes an E3 ubiquitin ligase that negatively regulates grain width and weight via the proteasome pathway	[48,49]
	*GW5*	Encodes a calmodulin-binding protein that negatively regulates grain width and weight through the brassinosteroid (BR) signaling pathway	[50]
	*GW8*	Orchestrates grain size, grain shape, and rice quality	[24]
	*GLW7*	Positively regulates hull cell size, leading to increased grain length and weight in rice	[51]
	*GL7*	A major-effect QTL for increased grain length-to-width ratio and reduced chalkiness	[52]
	*WTG1*	Encodes an Otubain-like protease that regulates rice grain size and shape through its effect on hull cell expansion	[53]

### 2.2. Environmental Factors Influencing Grain Quality

#### 2.2.1. Temperature Stress

**Figure 2:**
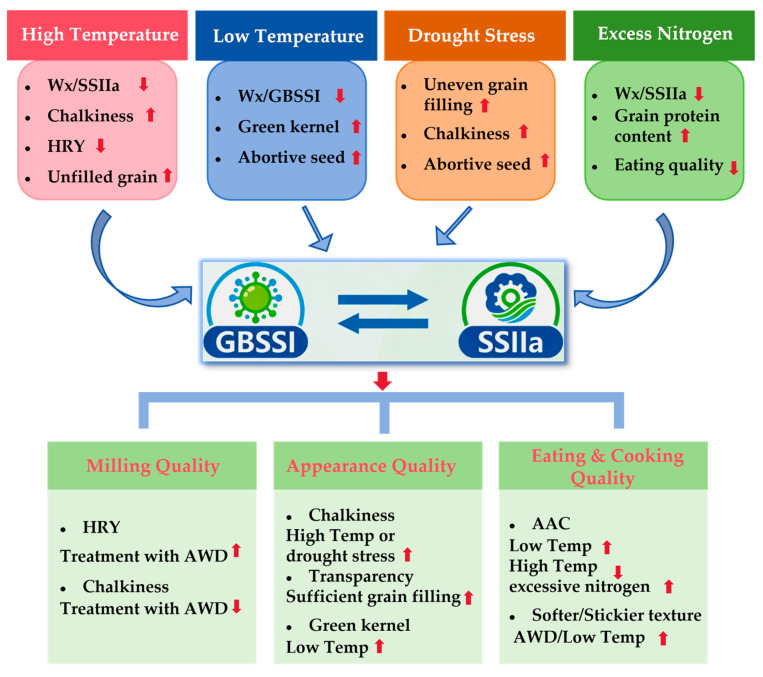
Simplified regulatory network illustrating the major environmental factors influencing rice grain quality during the grain-filling stage. This schematic diagram depicts the key regulatory pathways through which high temperature, low temperature, drought stress, alternate wetting and drying (AWD), and excess nitrogen fertilization affect rice grain quality (Temp: temperature).

Environmental factors play a decisive role in rice grain quality formation (Figure 2): they directly affect starch and protein deposition, endosperm structure, and grain appearance, while interacting in complex ways with the varietal genotype and management to determine seasonal quality outcomes [54,55,56]. High temperature, particularly during post-flowering grain filling stage and high night temperature (HNT), is a major climatic factor degrading rice appearance and eating quality. Extensive field and controlled experiments demonstrate that grain-filling heat significantly increases chalkiness, reduces head rice yield and grain uniformity, and often compromises eating quality [21,57]. HNT disrupts starch deposition, modifying GT and RVA profiles, typically lowering GT and transparency [58,59].

Heat stress inhibits or imbalances starch synthase complex activity and assembly (affecting *GBSSI/Wx*, *ALK*, SBE enzyme activity and expression), modifying AC, branch-chain length distribution, and starch granule arrangement, resulting in endosperm voids and chalkiness. Additionally, heat induces protein synthesis and heat shock protein/folding demands, elevating grain protein ratios and further impacting texture. Genotypic variation is pronounced: certain *Wx* or *SSIIa* alleles perform worse under heat, emphasizing gene-temperature interactions in heat sensitivity [6,60,61].

Low-temperature stress, particularly during the sensitive grain-filling stage, severely compromises rice grain quality. It disrupts key physiological processes, resulting in incomplete grain development and maturation. This directly results in an increase in the percentage of green kernels and abortive seeds due to impaired starch and protein deposition in the endosperm. Consequently, these immature and structurally weak grains are highly susceptible to breakage during milling, resulting in a significant reduction in head rice yield, which severely undermines milling quality [62,63,64]. Regarding cooking and eating quality, low-temperature stress often exerts an opposite effect to that of high-temperature stress. It typically results in a decrease in apparent amylose content, a primary determinant of cooked rice hardness. This reduction in amylose content is linked to the suppressed activity of key starch synthesis enzymes, especially granule-bound starch synthase (GBSS) encoded by the *Wx* gene [65]. While lower amylose content generally contributes to a softer and stickier texture—a preferred trait in some markets—the overall eating quality under severe cold stress is often offset by the undesirable appearance and high percentage of immature grains. Furthermore, cold stress alters the amylopectin chain-length distribution and can lead to inconsistent gel consistency, creating unpredictable cooking results [66].

#### 2.2.2. Water Management and Drought Stress

Water management is a crucial agricultural practice that significantly influences rice grain quality, particularly milling and appearance attributes [67,68,69]. Water scarcity is one of the primary abiotic stresses limiting global rice production. Beyond significantly reducing yield, drought stress profoundly alters the final quality of rice by affecting a series of physiological and biochemical processes, including grain filling, substance translocation, and metabolism, encompassing milling, appearance, cooking and eating quality, and nutritional aspects [70,71]. Milling quality, particularly head rice yield (HRY), demonstrates particular sensitivity to water stress during critical growth stages [72,73]. Moderate water deficit during grain filling typically increases HRY by 3–8% compared to continuous flooding, primarily through improved panicle architecture and enhanced remobilization of pre-stored carbohydrates [74]. However, severe water stress at any reproductive stage causes irreversible damage, reducing HRY by up to 15% [75]. Systematic reviews and meta-analyses show that filling-stage drought reduces head rice yield, increases chalkiness, alters amylose content and branch ratios, and compromises eating quality [76,77]. Drought stress, particularly during the grain-filling stage, disrupts starch and protein synthesis and deposition in the endosperm [75]. This leads to inadequate and uneven grain filling, resulting in increased chalkiness, significantly elevating the chalky grain rate and chalkiness degree, which severely compromises the appearance quality and commercial value of rice [78].

Alternate wetting and drying (AWD) has been recognized as a water-saving technology in rice production systems [79,80]; however, pre- and post-flowering AWD may induce changes in yield, quality and aroma biosynthesis in fragrant rice [81]. The interaction between water management and temperature further influences quality outcomes. Under high temperature conditions, maintained flooding provides evaporative cooling that mitigates heat-induced chalkiness, whereas under moderate temperatures, AWD generally produces superior quality [82]. This interaction emphasizes the need for context-specific water management strategies tailored to local climate conditions [83]. Research has demonstrated that controlled AWD implementation during mid-grain filling increased HRY by optimizing grain filling uniformity and enhancing endosperm structural integrity through modified starch deposition patterns [84,85].

#### 2.2.3. Soil Properties and Nutrient Management

Soil characteristics and fertilization practices constitute fundamental determinants of rice grain quality, influencing both physical attributes and chemical composition through complex soil–plant interactions [9,86,87]. These factors influence nutrient availability, root development, and physiological processes that ultimately define grain quality parameters. Soil characteristics directly influence grain quality [88,89,90,91]. Heavy clay soils with poor drainage can induce hypoxia during critical growth stages, hindering nutrient uptake and assimilate translocation to developing grains [92]. Soil compaction reduces head rice yield, primarily through impaired nutrient uptake that weakens endosperm cell walls. Conversely, well-structured loamy soils with optimal bulk density promote uniform grain filling and reduce chalkiness through balanced water and nutrient availability [93]. Soil pH represents another critical factor affecting mineral availability and grain composition [94,95]. Acidic soils (pH < 5.5) increase cadmium toxicity while reducing phosphorus and molybdenum availability, conditions that elevate grain chalkiness and reduce milling recovery [96].

Nitrogen (N) fertilization exhibits complex relationships with grain quality [97,98,99]. While essential for yield formation, excessive nitrogen application consistently degrades eating quality through multiple mechanisms [100,101]. Elevated N availability during grain filling increases protein content by 15–30%, primarily through upregulated synthesis of glutelins and prolamins. This increase in protein content correlates strongly with harder-cooked rice texture and reduced consumer acceptability [102,103]. Recent findings reveal that high N levels suppress the expression of starch biosynthesis genes (particularly *GBSSI* and *SSIIa*) while upregulating storage protein genes, resulting in an unfavorable starch-to-protein ratio for eating quality [104,105]. The timing of N application is crucial for quality optimization. Basal and tillering stage applications primarily affect yield components, while panicle initiation and grain filling applications directly influence quality parameters [106,107]. Research demonstrates that splitting N application maintains yield while reducing protein content by 12% compared to conventional two-split applications, significantly improving eating quality scores [108,109].

Specific micronutrients provide targeted approaches for quality enhancement. Silicon (Si) fertilization has proven to be particularly valuable for both general quality improvement and safety enhancement [9]. Previous research demonstrates the effects of different Si application methods on cadmium (Cd) accumulation and rice yield in two typical paddy soils in Chongqing, revealing that foliar spray, especially with Se-containing Si fertilizer, most effectively reduces grain Cd content and improves nutritional quality while enhancing productivity, providing a practical strategy for safe rice production in Cd-contaminated areas [110]. Research has investigated the effects of Si on the yield and quality of fragrant rice, focusing on its regulatory mechanisms in the accumulation of the key aroma compound 2-acetyl-1-pyrroline (2AP), by analyzing proline content and the expression of related synthesis and transport genes (including *Badh2*, *DAO*, *OAT*, *ProDH*, and *P5CS*), to explore the potential of Si as a beneficial nutrient in breeding high-quality aromatic rice [111]. Research has studied the mitigating effect of Si fertilization on Cd toxicity in both selenium-enriched (Z3055B) and non-selenium-enriched (G46B) rice genotypes, demonstrating that Si application enhances growth, yield, and grain quality by reducing Cd accumulation and improving selenium content [112]. Additionally, other studies suggest that zinc supplementation and light intensity affect 2-acetyl-1-pyrroline (2AP) formation in fragrant rice [113].

#### 2.2.4. Post-Harvest Handling

Rice quality—encompassing appearance, milling, cooking, and sensory attributes—is governed by the interplay between genetic potential and post-harvest management. Even elite varieties with superior genetic backgrounds are susceptible to irreversible quality deterioration when subjected to improper harvesting, drying, storage, or milling, ultimately compromising market value and consumer acceptance. Harvest timing is critical for preserving quality. For most *indica* and *japonica* rice, harvesting at 20–24% moisture content minimizes grain fissuring, chalkiness, and bran discoloration, while stabilizing apparent amylose content and gel consistency [114]. Delayed harvesting accelerates grain aging, increases fat acidity, and reduces transparency, leading to significant deterioration in eating quality [115]. Mechanical harvesting at optimal maturity also reduces physical grain damage, benefiting subsequent processing.

Drying is the most influential post-harvest operation affecting milling quality. Low-temperature (35–40 °C) uniform drying safely reduces paddy moisture to 13–14%, avoiding moisture-gradient-induced cracking. Recirculating dryers outperform traditional sun drying in preserving head rice yield and grain transparency [116], and post-drying tempering further alleviates fissuring and improves milling efficiency. Storage conditions largely determine long-term quality stability. Hermetic storage effectively suppresses pests, microbial activity, and moisture fluctuations, preserving apparent amylose content, gelatinization temperature, and sensory freshness. Conventional open storage accelerates lipid oxidation and flavor deterioration, whereas eco-friendly radiofrequency treatment offers effective pest control without compromising quality [117]. Gentle multi-stage milling maintains grain integrity, nutritional value, and appearance, whereas excessive polishing increases breakage and nutrient loss. Implementing precise post-harvest management is essential to fully realize the genetic quality potential of rice.

## 3. Conclusions

Rice grain quality is a multifaceted trait governed by a complex interplay of genetic, environmental, and management factors. Genetic architecture provides the foundational framework, with key genes such as *Wx*, *ALK*, *Chalk5* and *GS3/GW* families determining the potential for starch composition, gelatinization properties, chalkiness, and grain dimensions. However, the expression of this genetic potential is highly plastic and strongly influenced by environmental conditions. Abiotic stresses, particularly elevated temperatures during grain filling and water deficit, consistently reduce milling yield, increase chalkiness, and alter starch and protein profiles, thereby impairing appearance, cooking, and eating quality. Conversely, optimized agronomic practices, including precise water management (e.g., AWD), balanced and split N fertilization, and strategic micronutrient (e.g., silicon) application, can mitigate these adverse effects and potentially enhance specific quality parameters. The integration of post-harvest handling further determines the final quality delivered to consumers. This synthesis emphasizes that high and stable rice quality in variable conditions cannot rely on genetics or management alone. It requires an integrated approach that synergizes resilient genetic backgrounds, climate-smart field management, and careful post-production processing.

## 4. Prospect

Future research and innovation should focus on developing integrated and sustainable strategies to enhance rice grain quality resilience. Several promising frontiers emerge: (1) Next-generation breeding for quality resilience: Leveraging advanced genomic tools (e.g., CRISPR-based gene editing, genomic selection) to pyramid favorable alleles for multiple quality traits while incorporating climate resilience genes. A key target is developing varieties with “built-in” stability—genotypes in which quality-related pathways (e.g., starch biosynthesis, protein deposition, endosperm development) are less sensitive to fluctuations in temperature, water, and nitrogen. Exploring natural variation and engineered alleles of master regulators (e.g., transcription factor complexes like NF-YB1–YC12–bHLH144) that coordinate multiple quality-related processes under stress holds great promise. (2) Precision agronomy for targeted quality enhancement: Moving beyond uniform field management to dynamic, sensor-based precision agronomy. This involves real-time monitoring of canopy microclimate, soil moisture, and plant nitrogen status to tailor irrigation, fertilization, and even harvest timing. Developing models that predict quality outcomes based on genotype × environment × management (G × E × M) interactions will be crucial for providing decision-support tools to farmers to optimize both yield and quality. (3) Synergistic nutrient and soil health management: Further investigation into the role of microbiome-plant interactions in shaping grain quality under various soil and nutrient regimes is needed. Optimizing integrated nutrient management (INM) packages that combine organic amendments, silicon, zinc, and other beneficial elements to not only improve yield and quality but also reduce heavy metal accumulation (e.g., cadmium) and enhance nutritional safety (e.g., selenium) will be vital for sustainable production. (4) Post-harvest technology integration: Enhancing the quality chain requires innovation in adaptive post-harvest technologies. Research should focus on developing drying and storage protocols that are responsive to the initial quality status of harvested grains (influenced by pre-harvest conditions) to minimize fissuring and preserve aroma, nutritional content, and cooking characteristics. (5) Systems-level modeling and digital agriculture: Finally, a systems biology approach integrating omics data (genomics, transcriptomics, proteomics, metabolomics) with physiological and agronomic data is essential to fully decode the G × E × M nexus. This knowledge should be integrated into digital agriculture platforms, enabling the design of region-specific, climate-resilient rice production systems that reliably deliver high-quality grain to meet diverse consumer preferences and market demands in the face of global change. By converging advances in molecular biology, data science, and sustainable agronomy, the future of rice quality improvement lies in creating robust systems that ensure nutritional security, economic viability, and resilience to environmental challenges.

## Data Availability

The data and related conclusions presented in this article are all derived from https://pubmed.ncbi.nlm.nih.gov/ (accessed on 26 January 2026).
